# The role of wingbeat frequency and amplitude in flight power

**DOI:** 10.1098/rsif.2022.0168

**Published:** 2022-08-24

**Authors:** Krishnamoorthy Krishnan, Baptiste Garde, Ashley Bennison, Nik C. Cole, Emma-L. Cole, Jamie Darby, Kyle H. Elliott, Adam Fell, Agustina Gómez-Laich, Sophie de Grissac, Mark Jessopp, Emmanouil Lempidakis, Yuichi Mizutani, Aurélien Prudor, Michael Quetting, Flavio Quintana, Hermina Robotka, Alexandre Roulin, Peter G. Ryan, Kim Schalcher, Stefan Schoombie, Vikash Tatayah, Fred Tremblay, Henri Weimerskirch, Shannon Whelan, Martin Wikelski, Ken Yoda, Anders Hedenström, Emily L. C. Shepard

**Affiliations:** ^1^ Department of Biosciences, Swansea University, Swansea SA1 8PP, UK; ^2^ School of Biological, Earth and Environmental Sciences, University College Cork, Cork T23 N73 K, Ireland; ^3^ British Antarctic Survey, Natural Environment Research Council, Cambridge, UK; ^4^ Durrell Wildlife Conservation Trust, La Profonde Rue, Jersey JE3 5BP, Jersey; ^5^ Department of Natural Resources Sciences, McGill University, Sainte-Anne-de-Bellevue, Quebec, Canada; ^6^ Biological and Environmental Sciences, University of Stirling, Stirling FK9 4LA, UK; ^7^ Departamento de Ecología, Genética y Evolución and Instituto de Ecología, Genética Y Evolución de Buenos Aires (IEGEBA), CONICET, Pabellón II Ciudad Universitaria, C1428EGA Buenos Aires, Argentina; ^8^ Diomedea Science – Research and Scientific Communication, 819 route de la Jars, 38 950 Quaix-en-Chartreuse, France; ^9^ Graduate School of Environmental Studies, Nagoya University, Furo-cho, Chikusa-ku, Nagoya 464-8601, Japan; ^10^ Centres d'Etudes Biologiques de Chizé – CNRS, Villiers-en-Bois, France; ^11^ Department of Migration, Max Planck Institute of Animal Behavior, Radolfzell, Germany; ^12^ Instituto de Biología de Organismos Marinos (IBIOMAR), CONICET, Boulevard Brown, 2915, U9120ACD, Puerto Madryn, Chubut, Argentina; ^13^ Max Planck Institute for Ornithology, Seewiesen, Germany; ^14^ Department of Ecology and Evolution, University of Lausanne, Building Biophore, 1015 Lausanne, Switzerland; ^15^ FitzPatrick Institute of African Ornithology, University of Cape Town, Rondebosch, South Africa; ^16^ Mauritian Wildlife Foundation, Grannum Road, Vacoas 73418, Mauritius; ^17^ Centre for the Advanced Study of Collective Behaviour, University of Konstanz, 78457 Konstanz, Germany; ^18^ Department of Biology, Centre for Animal Movement Research, Lund University, Lund, Sweden

**Keywords:** energy expenditure, accelerometry, kinematics, bio-logging, movement ecology

## Abstract

Body-mounted accelerometers provide a new prospect for estimating power use in flying birds, as the signal varies with the two major kinematic determinants of aerodynamic power: wingbeat frequency and amplitude. Yet wingbeat frequency is sometimes used as a proxy for power output in isolation. There is, therefore, a need to understand which kinematic parameter birds vary and whether this is predicted by flight mode (e.g. accelerating, ascending/descending flight), speed or morphology. We investigate this using high-frequency acceleration data from (i) 14 species flying in the wild, (ii) two species flying in controlled conditions in a wind tunnel and (iii) a review of experimental and field studies. While wingbeat frequency and amplitude were positively correlated, *R*^2^ values were generally low, supporting the idea that parameters can vary independently. Indeed, birds were more likely to modulate wingbeat amplitude for more energy-demanding flight modes, including climbing and take-off. Nonetheless, the striking variability, even within species and flight types, highlights the complexity of describing the kinematic relationships, which appear sensitive to both the biological and physical context. Notwithstanding this, acceleration metrics that incorporate both kinematic parameters should be more robust proxies for power than wingbeat frequency alone.

## 1. Introduction

Factors affecting the energetic costs of flight can have a profound influence on the ecology and behaviour of birds, with flight conditions affecting the location of migratory flyways, and in particular cases, breeding success [1,2]. Yet at fine scales, disentangling the impact of the biological and physical environment on flight costs can be challenging, given that a range of factors often vary simultaneously. These include the topography birds are flying over, individual position within a flock [3,4] and social context [5,6], as well as factors that vary over longer timescales including the birds' immunological state [7], and physical factors such as wind speed, turbulence and air density [8–10]. High-frequency data from animal-attached loggers have proved powerful in this regard, as the signal from onboard accelerometers can be used to quantify second-by-second changes in wingbeat frequency [11–13], and potentially other kinematic parameters [14].

Power varies in a U-shaped fashion with flight speed (specifically the airspeed) for most flying birds [15–19], and wingbeat frequency seems to follow the same trend, although it is not always pronounced [6,19–23]. This explains why wingbeat frequency has been used as a proxy for flight costs in a range of ecological studies (e.g. [6,14]). However, wingbeat frequency also has limitations as a proxy for power requirements, because studies by Hedrick *et al*. [21] and Tobalske *et al*. [19] have shown that the minimum wingbeat frequency does not always coincide with the minimum power speed. In fact, it can occur at over twice the minimum power speed, which demonstrates that other kinematic parameters, such as wingbeat amplitude, stroke-plane angle and span ratio, can have an important role in modulating power output [24–27].

The major determinants of the aerodynamic power output of a flapping wing are the wingbeat frequency (*f*) and amplitude (*A*). In flapping flight, the resultant aerodynamic forces (lift, drag and thrust) acting on the wing are predominantly determined by the flow over each wing section at each time instant [28]. This is the combination of the flow due to the forward velocity of the bird and the flapping motion of the wing (wing velocity). The flow over the wing section can be controlled by the wing velocity, which solely depends on the wingbeat frequency and wingbeat amplitude [18]. The aerodynamic forces exerted on the wings are proportional to the square of the velocity, and the mechanical power output is proportional to the cube of the velocity [18]. Therefore, while the total resultant aerodynamic forces can be modulated by varying the wing planform and angle of attack during flight, modulating the flow velocity over each wing section has the major effect. The power can be shown to be proportional to the cube of both amplitude and frequency, if the product of wingbeat amplitude and frequency is substituted for velocity (as they both scale the same with velocity [29]):

$$\mathrm{Power}∼A^{3}f^{3}.$$

Despite the importance of both wingbeat frequency and amplitude for overall power output, an overview of the scenarios under which birds modulate one or the other parameter is lacking. Indeed, examples from the literature suggest that the relationship may not be straightforward. Some studies show that birds vary their power output with little to no change in wingbeat frequency [30–32], whereas others report that wingbeat frequency varies with the power output while the amplitude is unaltered [20]. It is, therefore, unclear whether birds vary frequency or amplitude to modulate power according to their flight mode (e.g. hovering, climbing, manoeuvring or level flight) or morphology.

Power can theoretically be modulated either by a contribution from both wingbeat frequency and amplitude, or by changes to one or the other. What is clear is that a proxy for flight power should ideally integrate information on wingbeat frequency and amplitude to be widely applicable. Two related proxies for energy expenditure have been proposed using data from body-mounted accelerometers, both of which integrate information on stroke frequency and signal amplitude. Dynamic body acceleration (DBA) was proposed in 2006 as a metric that captures whole-body acceleration [33,34], and has been shown to vary with the energy expended by free-living auks [35] and cormorants [7] in flight. However, the precise relationship between the DBA signal and wingbeat kinematics is unknown. Spivey & Bishop [36] also established a theoretical framework of how body acceleration can be related to the biomechanical power output of flapping flight, using the root mean square values of heave and surge acceleration and wingbeat frequency. This assumes that the amplitude of the dorsoventral or ‘heave’ accelerometer measurements vary with the wingbeat amplitude [6]. However, similar to DBA, the relationship between body and wing motions, and how they covary over a wingbeat cycle, has not been established.

In this study, we examine the outlook for acceleration-based proxies for power use in flapping flight across species and contexts. Specifically, we (i) test how the output of body-mounted accelerometers varies with wingbeat amplitude, using a novel methodology, and (ii) assess whether birds preferentially use wingbeat frequency or amplitude to modulate their power output (or speed, as a related response) according to (a) their body mass or morphology and (b) their flight mode. We address this by reviewing the experimental literature, where wingbeat kinematics have largely been quantified using high-speed video, and by conducting further trials, where we equip 14 species of bird with body-mounted accelerometers to monitor their flight behaviour in the wild.

## 2. Methods

### 2.1. Wind tunnel trials*:* does the acceleration signal vary with wingbeat amplitude?

Movement of the wings results in movement of the body in the same axis. Greater wingbeat amplitudes should result in greater vertical accelerations of the body for a fixed wingbeat frequency. We examined these relationships using a body-mounted accelerometer and magnetometer, and a small neodymium boron magnet attached to the leading edge of the wing [37]. The geomagnetic signal strength in each axis varied throughout the wingbeat cycle as a function of the angle and distance to the magnet. We, therefore, calculated the vector sum from all three magnetometer channels, which varied solely with the distance to the magnet, giving a clear peak per wingbeat cycle when the magnet was closest to the sensor. This allowed us to assess how the vertical body acceleration varied in relation to the maximum vector sum from the magnetometer (as a proxy for wingbeat amplitude) within the same wingbeat cycle.

**Figure 1.**
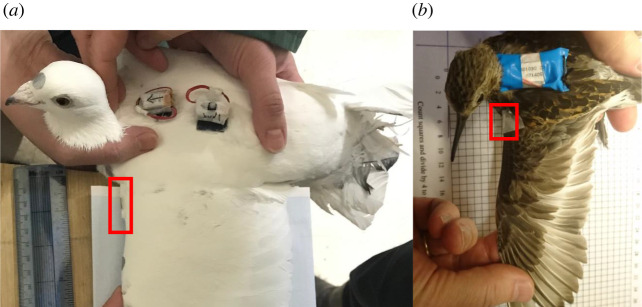
Set-up of the tag (DD; containing an accelerometer and magnetometer) and magnet (highlighted by the red rectangle) on (*a*) a pigeon and (*b*) a dunlin.

Data were collected from two species flying at a range of speeds in large, low turbulence wind tunnels. In one set of trials, two pigeons (*Columba livia*) were equipped with Daily Diary (DD) data loggers (Wildbyte Technologies, Swansea University, UK), sampling acceleration at 150 Hz and magnetic field strength at 13 Hz. Each pigeon was equipped with two units: one on the upper back and another on the lower back. The logger at the top of the back was positioned close to the magnet, whereas the logger on the lower back was sufficiently far from the magnet not to be influenced by it (as determined in preliminary tests). The second logger allowed us to control for the potential influence of changing geomagnetic field strength on the magnetometer output (due to changes in bird trajectory). Loggers had dimensions of 22 mm × 15 mm × 9 mm and a total mass that was less than 3% of the bird body mass (3.4 g per logger and battery). A cylindrical neodymium boron magnet (8 mm × 2 mm, 0.19 g) was taped to the leading edge of the wing, close to the wing root (figure 1*a*). Both the loggers and the magnet were attached with micropore tape. Pigeons were flown at speeds between 12 and 18 m s^−1^. Experiments were performed between 25 January 2019 and 1 February 2019 in the wind tunnel of the Max Planck Institute for Ornithology, Germany, under ethical approval Gz.: 55.2-1-54-2532-86-2015 granted by the government of Upper Bavaria (Sachgebiet 54—Verbraucherschutz, Veterinärwesen, 80538 München).

Further trials were conducted with a dunlin (*Calidris alpina*) in the wind tunnel at Lund University, Sweden, which has similar performance characteristics to the tunnel in Seewiesen [38]. A small neodymium magnet (4 mm × 2 mm, 0.02 g) was attached to the wing of the dunlin following the same procedure. A single unit logging tri-axial acceleration and magnetic field strength at 100 Hz (Technosmart Europe) was attached to the back of the dunlin with a backpack harness (figure 1*b*). The logger was 16 mm × 24 mm × 12 mm and weighed 2.6 g, equivalent to 4.8% of the bird's body mass. The dunlin was flown at a range of speeds for less than 10 min. Ethical permission for all wind tunnel trials was obtained from Swansea University AWERB, permit no. 030718/66.

### 2.2. Variation in the amplitude–frequency relationship across species

Data from birds flying in the wind tunnel were combined with acceleration data from a further 12 species of free-flying birds (table 1) to examine relationships between wingbeat frequency and amplitude, and whether birds are more likely to use one parameter or the other to modulate their power output, according to their mass and morphology. Datasets were selected for inclusion according to whether tags were attached on the back, rather than the tail, to minimize the contribution of the angular motion of the bird to the acceleration signal, when the sensor is placed far from the centre of mass [47].

**Table 1.** Datasets in the study, along with the number of individuals tracked, body mass, wingspan and wing area, and the source of the morphometric data.

species	location	*N*	mass (g)	wingspan (m)	wing area (m^2^)	tag type	data from literature	source
Brünnich's guillemot *Uria lomvia*	Coats Island, Nunavut, Canada	13	949	0.727	0.069	Daily Diary	wings	Orben *et al*. [39]
common guillemot *Uria aalge*	Puffin Island, UK	6	1050	0.73	0.056	AxyTrek	mass, wings	Spear & Ainley [40]
northern fulmar *Fulmarus glacialis*	Saltee Islands, Ireland	3	778	1.12	0.106	Daily Diary	wings	Warham [41]
pigeon *Columba livia*	Radolfzell, Germany	9	456	0.647	0.064	Daily Diary	none	measured directly
red-tailed tropicbird *Phaethon rubricauda*	Round Island, Mauritius	10	820	1.115	0.117	Daily Diary	none	measured directly
great frigatebird *Fregata minor*	Europa Island	3	1113	2.084	0.365	Daily Diary	none	measured directly
black-legged kittiwake *Rissa tridactyla*	Middleton Island, Alaska, USA	3	387	0.965	0.101	Daily Diary	wings	Pennycuick [42] (*n* = 2)
imperial cormorant *Leucocarbo atriceps*	Punta Leon, Argentina	5	2400	1.13	0.183	Daily Diary	mass, wings	Quintana *et al*. [43], Spear & Ainley [40] (*n* = 1)
western barn owl *Tyto alba*	Switzerland	10	296	0.936	0.134	AxyTrek	none	measured directly
grey-headed albatross *Thalassarche chrysostoma*	Marion Island, South Africa	5	3290	2.186	0.348	Daily Diary	mass, wings	Phillips *et al*. [44] (*n* = 1)
wandering albatross *Diomedea exulans*	Marion Island, South Africa	6	8500	3.01	0.583	Daily Diary	mass, wings	Pennycuick [42]; Pennycuick [18]
streaked shearwater *Calonectris leucomelas*	Awashima Island, Japan	5	503	1.119	0.126	Daily Diary	wings	Shirai *et al*. [45]
dunlin *Calidris alpina*	Sweden	1	55	0.334	0.014	Axy XS	mass, wings	Hentze [46]
northern gannet *Morus bassanus*	Saltee Islands, Ireland	10	2856	1.85	0.262	Axy	wings	Spear & Ainley [40] (*n* = 1)

Morphological parameters including wing loading, wingspan, wing area and body mass were either measured directly and averaged (following [18]) or taken from the literature (table 1). We used wingspan rather than aspect ratio because there is a framework linking the former to wingbeat kinematics [18]. In order to assess the role of wing loading independently from body mass, we calculated the residuals of the linear regression between log(wing loading) and log(body mass) [48].

All birds flying in the wild were equipped with tags recording tri-axial acceleration at 40 Hz (except common guillemots and gannets, where the sampling rate was 50 Hz, and pigeons, where it was 180 Hz). An examination of accelerometer data revealed some slight variation in sampling rate between logger types (up to 3 Hz), which was accounted for in the calculation of wingbeat frequency. Tags were attached to the back feathers using Tesa tape [49] in all species apart from pigeons, where tags were attached via Velcro strips glued to the back feathers [3,50]. The total mass of the tag, including housing and attachments, was under 5% of bird body mass and 3% in most cases. See electronic supplementary material, table S1, for details of ethical permissions.

**Figure 2.**
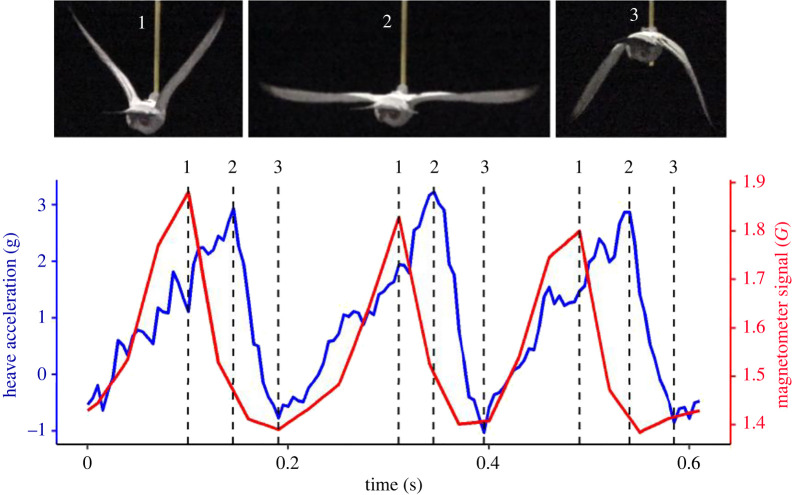
Comparison of the accelerometer (blue) and magnetometer (red) signals in the heave axis for three wingbeats from a pigeon flying in a wind tunnel at 15 m s^−1^. (1) Peaks in the magnetometer signal correspond to the start of the downstroke (a smaller acceleration peak is sometimes evident at the same time), (2) peaks in the heave acceleration occur in the middle of the downstroke and (3) troughs in the magnetometer signal occur at the end of the downstroke. Images from the corresponding wingbeat cycle were captured using a Sony PXW-Z150 camera recording at 120 Hz, which was synchronized with the onboard logger by moving the equipped bird in view of the camera and a clock showing the logger time.

Episodes of flapping flight were identified visually from the acceleration data [51]. Only periods of consistent flapping, with no interruption or rapid changes in amplitude, were selected for the analysis of both wind tunnel and wild data, irrespective of the species. Wingbeat frequency and heave amplitude (amplitude of the vertical body acceleration within a wingbeat) were quantified using the following approach, which enabled the estimation of the period of individual wingbeats. Peaks in heave acceleration associated with the downstroke (figure 2) were identified by smoothing raw heave values over three to five datapoints for all species except the guillemots, which did not require smoothing as their high wingbeat frequency resulted in a relatively clean signal. A second-order derivative was then applied to identify the positive-to-negative turning points. Peaks were marked when the differentials exceeded a threshold within five points of the turning point. Thresholds were manually selected for each flight bout so that they only captured wingbeat peaks, as characterized by high heave accelerations (around 2 g). The section between each marked peak was considered as one wingbeat cycle and used to determine the wingbeat period (frequency). The wingbeat frequency of the dynamic soaring birds (birds that extract energy by flying through the wind shear in the atmosphere) represents the frequency during the flapping period. The heave amplitude was calculated as the difference between the highest and lowest heave values within the wingbeat. Peak identification was conducted in R, v. 4.0.2 [52] using user-defined functions for Brünnich's guillemot, common guillemots, pigeons (homing flights only) and tropicbirds. All other data were processed using custom developed software DDMT (Wildbyte Technologies).

Filters were applied to remove unrealistic wingbeat frequencies. Low outliers were identified during short sections of non-flapping flights that were not excluded during the previous steps. High outliers were also recorded and were probably caused by false peak identification due to rapid manoeuvres. Filtered data were used to estimate final wingbeat frequencies, taken as the average over 10 consecutive wingbeats for wild data (which sometimes occurred in two flapping bouts for albatrosses) and five wingbeats for wind tunnel data (where the total wingbeats available from consistent flights was lower). Heave amplitude was also averaged over the same interval.

Finally, a simulation confirmed that our ability to estimate signal amplitude across species with variable wingbeat frequencies was not influenced by the sampling frequency (electronic supplementary material).

### 2.3. Variation in wingbeat kinematics with climb rate and airspeed

First, we examined how wingbeat frequency and signal amplitude varied in relation to airspeed for a pigeon flying in the wind tunnel (for which we had reliable records of airspeed). Then we assessed how pigeons, barn owls and tropicbirds varied their wingbeat frequency and amplitude in relation to airspeed and climb rate in the field. These datasets were selected due to the relatively high GPS sampling frequency (1 Hz for pigeons and barn owls, and once per minute for tropicbirds). Airspeed was estimated from the GPS-derived groundspeed and the wind vector [18], as recorded by a portable weather station (Kestrel 5500 L, Kestrel Instruments, USA) mounted on a 5 m pole (see [3]). The weather station was positioned at the pigeons' release site, and at the highest point of Round Island (280 m.a.s.l.) in the case of the tropicbirds. For barn owls, weather data were collected from weather stations located near the nest sites. Altitude was calculated from barometric pressure recorded by the DD (at 4 Hz) in the case of the pigeons and tropicbirds, adjusted for daily changes in sea level pressure [3] and climb rate was calculated as the difference between consecutive values of altitude smoothed over 2 s. GPS altitude was used for the barn owls.

Airspeed, climb rate, wingbeat frequency and heave amplitude were averaged over 10 wingbeats for the pigeons and barn owls, and over 1-min intervals for the tropicbirds (to match the airspeeds). For each interval (10 wingbeats or 1 min) the proportion of level flapping flight was calculated, and only intervals with greater than or equal to 80% level flapping flight were included in the analysis.

Periods of level flapping flight were selected for the airspeed analysis, taking data where the rate of change of altitude was greater than −0.2 and less than 0.2 m s^−1^. To minimize the variation in airspeed in the climb rate analysis, we excluded data with airspeeds higher or lower than the overall mean ± 1 s.d.

### 2.4. Statistical analysis

We used linear models to examine whether the peak heave acceleration increased with the peak magnetometer vectorial sum (as a proxy for wingbeat amplitude) for both dunlin and pigeon wind tunnel flights. We also used linear models to assess whether the heave amplitude varied with wingbeat frequency, using separate models for wind tunnel and wild flights.

To test whether birds varied their wingbeat amplitude to a greater extent than their wingbeat frequency in relation to climb rate and airspeed, we ran separate linear mixed-effects models (LMMs) per species (tropicbirds, barn owls and pigeons). These models included wingbeat amplitude as the response variable, expressed as a function of wingbeat frequency and the effect of either airspeed or climb rate on the slope of this relationship (the interaction between wingbeat frequency and either climb rate or airspeed). A positive interaction would indicate that birds increased their amplitude more than frequency to increase speed/climb rate, while a negative relationship would indicate that they modulate wingbeat frequency more than amplitude. Individual was included as a random factor to account for uncontrolled variation relating to morphology and motivation (only one trip per bird was included). A continuous-time first-order autoregressive correlation structure was included in all models.

To investigate whether morphology affected the degree to which birds varied their wingbeat frequency, we calculated the coefficient of variation for the wingbeat frequency for each species, with the prediction that groups such as auks, with high wing loading, would be constrained in their range of frequencies. We did not run this analysis for the signal amplitude data, as the signal magnitude (and how this varies, e.g. with flight speed) might be influenced by factors including device location [47]. We used linear models and Pearson's product-moment correlation tests to see how the species-specific coefficients of variation (used as response variables) varied with wingspan, body mass, and residual wing loading. Note that pigeon flights recorded in the wind tunnel were not used in this analysis as free flight data had been recorded for pigeons. The dunlin flights were included. All statistical analyses were performed using R v. 4.0.2. LMMs were performed using the package ‘nlme’ (R v. 3.1-151 [53]). Model selection was performed using the package ‘MuMIn’ (R v. 1.43.17 [54]), and the distribution of residuals was tested using ‘fitdistrplus’ (R v. 1.1-5 [55]).

## 3. Results

### 3.1. Wind tunnel trials*:* does the acceleration signal vary with wingbeat amplitude?

Pronounced cyclic changes in the magnetometer signal were evident through the wingbeat cycle for both species that were flown in the wind tunnel (figure 2) due to the changing magnetic field strength driven by the small magnet attached to the leading edge of the wing. The magnetometer signal was highest at the start of the downstroke when the distance between the magnet and the transducer was at a minimum, and it decreased as the downstroke progressed, until the magnet was farthest from the logger at the end of the downstroke (figure 2). By contrast, the maximum heave acceleration occurred mid-downstroke when the wing traversed the body, corresponding to the point of maximal lift generation [56,57]. The magnetometer signal, therefore, varied with the wing displacement rather than wing (and body) acceleration, explaining why the peaks in magnetic and acceleration signals were offset from each other.

**Figure 3.**
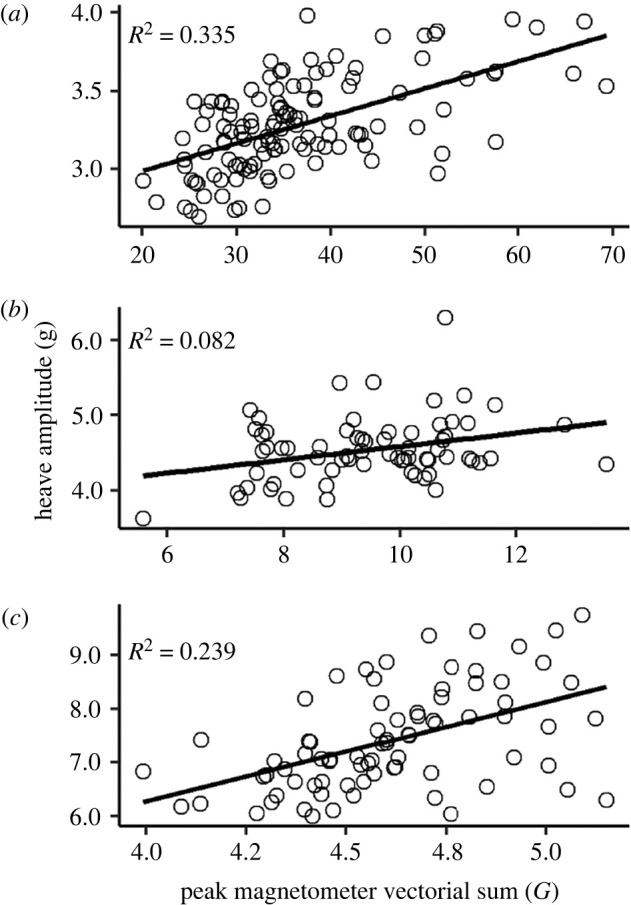
The heave amplitude increased with the maximum magnetometer vectorial sum within wingbeat cycles for (*a*) a dunlin and (*b,c*) two pigeons flying in wind tunnels across a range of flight speeds. The variation in absolute values from the magnetometer will vary due to the position of the magnet on the wing and its distance to the body-mounted magnetometer. The amplitude of the heave signal is influenced by the position of the back-mounted logger.

Nonetheless, we found a positive linear relationship between heave amplitude and the peak magnetometer vectorial sum in both species (pigeons: estimate = 1.253, s.e. = 1.02, *t*-value = 5.151, *p* < 0.001; dunlin: estimate = 2.639, s.e. = 0.085, *t*-value = 31.01, *p* < 0.001), showing that the body acceleration increases with wingbeat amplitude (figure 3).

### 3.2. Assessing the relationship between wingbeat amplitude and frequency

We then assessed how the wingbeat frequency and heave amplitude (as a proxy for wingbeat amplitude as established for pigeon and dunlin) covaried for different species. There was a positive, linear relationship between wingbeat frequency and heave amplitude in almost all species that flew in the wild (*n* = 13) and the wind tunnel (*n* = 2) (table 2). The exceptions were three of the four birds that use dynamic soaring: the northern fulmar, grey-headed albatross and wandering albatross. Nonetheless, most *R*^2^ values were relatively low, ranging from 0.001 to 0.38 (table 2).

**Table 2.** The relationship between heave amplitude and wingbeat frequency for 14 species flying in the wild and two species flying in controlled conditions.

species	signal amplitude (g)	wingbeat frequency (Hz)	slope	intercept	*p*-value	*R*^2^	total wingbeats
dunlin^a^	3.2 ± 0.3	13.2 ± 0.9	0.110	1.833	<0.001	0.112	73
pigeon^a^	6.0 ± 0.7	6.7 ± 0.4	0.893	1.337	<0.001	0.309	147
pigeon	3.7 ± 0.4	5.2 ± 0.5	0.189	2.713	<0.001	0.048	4858
barn owl	2.4 ± 0.4	4.4 ± 0.4	0.518	0.531	<0.001	0.162	134 919
common guillemot	2.5 ± 0.3	9.7 ± 0.6	0.206	0.541	<0.001	0.170	31 349
Brünnich's guillemot	1.3 ± 0.2	7.7 ± 0.5	0.180	−0.076	<0.001	0.195	122 598
imperial cormorant	1.1 ± 0.2	5.7 ± 0.2	0.190	0.044	<0.001	0.062	11 068
red-tailed tropicbird	1.8 ± 0.3	4.0 ± 0.3	0.527	−0.341	<0.001	0.151	174 190
black-legged kittiwake	2.0 ± 0.4	4.0 ± 0.2	0.998	−1.915	<0.001	0.383	21 767
great frigatebird	1.7 ± 0.3	2.6 ± 0.2	0.757	−0.213	<0.001	0.256	2805
streaked shearwater	1.4 ± 0.1	4.1 ± 0.3	0.018	1.315	<0.001	0.001	18 036
northern fulmar	1.3 ± 0.1	4.7 ± 0.3	−0.003	1.354	0.437	0.000	8505
grey-headed albatross	1.4 ± 0.1	3.1 ± 0.2	0.016	1.325	0.500	−0.001	590
wandering albatross	1.1 ± 0.1	2.8 ± 0.2	0.043	0.952	0.207	0.001	533
northern gannet	2.5 ± 0.6	3.9 ± 0.3	0.489	0.632	<0.001	0.051	15 410

^a^Wind tunnel studies.

**Figure 4.**
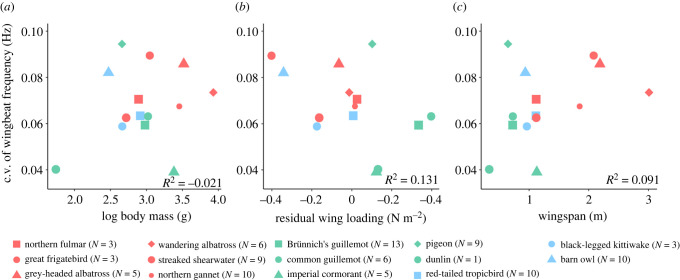
Variation in wingbeat frequency as a function of morphological parameters for 14 species: (*a*) body mass, (*b*) residual wing loading (where positive values indicate species with higher wing loading than expected for a given mass) and (*c*) wingspan. Birds with similar flights style are marked with the same colour: red represents specialist soaring fliers, green represents obligate flapping fliers and the blue indicates birds that use mix of flapping and soaring.

We then examined the coefficient of variation (c.v.) in wingbeat frequency, to assess whether this varied with bird mass or morphology. These coefficients were calculated by pooling data from all individuals of the same species to cover the various flight conditions (e.g. wind speeds) experienced across tracks. None of the correlations were significant, but there was an indication that the variation in wingbeat frequency was negatively correlated with the residual wing loading (figure 4) (Pearson's correlation: *ρ* = −0.445, *R*^2^ = 0.131, *p*-value = 0.111).

### 3.3. Do birds adjust different kinematic parameters to vary speed and climb rate?

Climb rate had a positive effect on the relationship between wingbeat frequency and amplitude in tropicbirds, demonstrating that birds increased their wingbeat amplitude to a greater extent in climbing flight (table 3). The same effect was seen in barn owls, although the *R*^2^ was low. We were not able to make any meaningful conclusion concerning pigeons flying in the wild as the fixed effects in the model explained only 1% of the variance in the response variable ($R_{m}^{2}=0.01$; table 3).

**Table 3.** Models of heave amplitude as a function of wingbeat frequency (WBF) and the interaction between wingbeat frequency and climb rate (*V_z_*) for red-tailed tropicbirds (*n* = 10), pigeons (*n* = 9) and barn owls (*n* = 10), using individual as a random factor.

	estimate	s.e.	*t*-value	*p*-value
tropicbirds ($R_{m}^{2}=0.50$, $R_{c}^{2}=0.66$)				
(intercept)	−2.275	0.056	−40.622	<0.001
WBF	1.014	0.011	91.817	<0.001
WBF: *V_z_*	0.018	0.001	13.301	<0.001
pigeons ($R_{m}^{2}=0.01$, $R_{c}^{2}=0.42$)				
(intercept)	3.882	0.132	29.524	<0.001
WBF	−0.053	0.018	−3.013	0.003
WBF: *V_z_*	−0.008	0.003	−3.256	0.001
barn owls ($R_{m}^{2}=0.28$, $R_{c}^{2}=0.65$)				
(intercept)	−0.615	0.0799	−7.7	<0.001
WBF	0.677	0.0045	150.3	<0.001
WBF: *V*_z_	0.048	0.0008	59.3	<0.001

**Figure 5.**
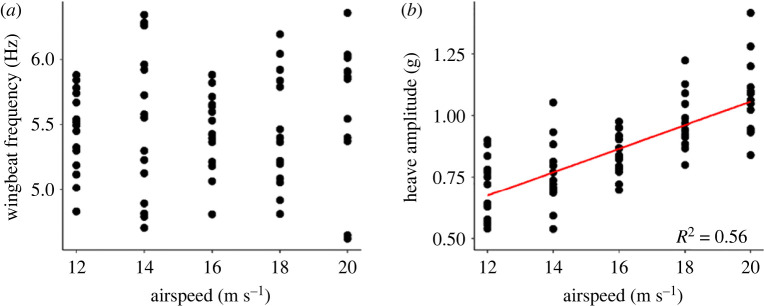
(*a*) Wingbeat frequency and (*b*) signal amplitude for a pigeon flying in a wind tunnel at a range of airspeeds. Each data point is an average of five consecutive wingbeats. Periods of consistent flight were selected for analysis.

Airspeed did not affect the relationship between wingbeat frequency and amplitude in tropicbirds (*p* = 0.164), barn owls (*p* = 0.546), or in pigeons, where the model explained only 3% of the variability in the response variable ($R_{m}^{2}=0.03$; see electronic supplementary material, table S2). By contrast, there was a clear increase in heave amplitude with airspeed for a pigeon flying in the wind tunnel (figure 5).

We found 22 studies where the relationship between wingbeat frequency, wingbeat amplitude and either mechanical power, speed or climb rate was quantified (table 4). Of these, 10 were performed with Passeriformes. Kinematic analyses were mostly conducted using high-speed cameras to quantify wingbeat frequency and amplitude for birds either flying in wind tunnels or flight chambers.

**Table 4.** Summary of studies assessing the relationship between wingbeat frequency, amplitude and mechanical power output.

species	method	flight mode	speed (m s^−1^)	remarks	source
pigeon *Columba livia*	field data—GPS and accelerometer measurements	level, ascending and descending flight, all while circling	10–18	*as speed increased*	Usherwood *et al*. [6]
				WBF—varied approx. U shaped	
				WBA—increased	
				*at constant speed, as power increased*	
				WBF—increased	
				WBA—decreased	
				*ascending flight*	
				WBF—increased	
				WBA—increased	
				*accelerating flight*	
				WBF—increased	
				WBA—increased	
pigeon *Columba livia*	platform—muscle force measurements and kinematic analysis with high-speed cameras	ascending, level and descending	1.4–3.9	*in different flight modes*	Tobalske & Biewener [30]
				WBF—did not vary significantly	
				WBA—decreased during take-off and prior to landing	
common starling *Sturnus vulgaris*	wind tunnel—respirometry masks and kinematics analysis with high-speed cameras	level flight	6–14	*as speed increased*	Ward *et al*. [27]
				WBF—increased (less significant)	
				WBA—increased (less significant)	
				power—increased	
Eurasian tree sparrow *Passer montanus*	experiments in flight chamber—kinematics analysis with high-speed cameras	vertical flight	—	*as maximum load lifted*	Wang *et al*. [32]
				WBF—no significant variation	
				WBF—no significant variation	
barn swallow *Hirundo rustica*	wind tunnel—energetic costs measured by DLW, and kinematics analysis is by video recordings	level flight	8–11.5	*as speed increased*	Schmidt-Wellenburg *et al*. [23]
				WBF—varied as U shaped	
				*as mass increased*	
				WBF—increased	
				power—increased	
blue tit *Cyanistes caeruleus*	flight inside a custom-built box—kinematics analysis with high-speed cameras	take-off	3.4	*as wing loading increased*	McFarlane *et al*. [58]
				WBF—decreased	
				WBA—did not vary	
				power—decreased	
				AR—increased	
thrush nightingale *Luscinia luscinia*	wind tunnel—PIV and kinematics analysis with high-speed cameras	level flight	5–10	*as speed increased*	Rosén *et al*. [25]
				WBF—no significant variation	
				WBA—no significant variation	
thrush nightingale *Luscinia luscinia*	wind tunnel—wingbeat frequency measured using a shutter stroboscope and video recording	level flight	5–16	*as mass increased*	Pennycuick *et al*. [22]
				WBF—increased	
				*as speed increased*	
				WBF—varied in U shape (less significantly)	
zebra finch *Taeniopygia guttata*	wind tunnel—kinematics analysis with high-speed cameras	intermittent flap-bounding flight	0–14	*as speed increased*	Tobalske *et al*. [59]
				WBF—increased (less significant)	
				WBA—decreased (significantly)	
zebra finch *Taeniopygia guttata*	surgical procedures to measure flight muscle activity	?	—	*as power increased*	Bahlman *et al*. [60]
				WBF—no significant effect	
				WBA—increased effectively	
zebra finch *Taeniopygia guttata*	wind tunnel—muscle *in vivo* pectoralis fascicle strain measurements, and kinematics by high-speed video recordings	level flight	0–14	*as speed increased*	Ellerby & Askew [20]
				WBF—varied approx. U shaped	
				WBA—increased only at hovering	
budgerigar *Melopsittacus undulates*	wind tunnel—muscle *in vivo* pectoralis fascicle strain measurements, and kinematics by high-speed video recordings	level flight	4–16	*as speed increased*	Ellerby & Askew [20]
				WBF—varied approx. U shaped	
				WBA—did not vary significantly	
cockatiel *Nymphicus hollandicus*	wind tunnel—*in vivo* pectoralis muscle length change measurements	level flight	0–16	*as speed increased*	Morris & Askew [61]
				power—increased (approx. U shaped)	
				WBF—reduced (highest at the lower range)	
cockatiel *Nymphicus hollandicus*	wind tunnel—*in vivo* surgical procedures and kinematics analysis with high-speed cameras	level flight	0–14	*as speed increased*	Hedrick *et al*. [21]; Tobalske *et al*. [19]
				WBF—reduced at lower speed and increased at higher speed (approx. U shaped)	
				power—varied (approx. U shaped)	
Eurasian teal *Anas crecca*	wind tunnel—wingbeat frequency measured using a shutter stroboscope and video recording	level flight	5–16	*as mass increased*	Pennycuick *et al*. [22]
				WBF—increased	
				*as speed increased*	
				WBF—varied in U shape (less significantly)	
black-legged kittiwake *Rissa tridactyla*	wild study—kinematics and airspeed data of commuting flights measured using GPS and accelerometer devices	flap–glide flight (predominantly flapping)	2–16	*as speed increased*	Collins *et al*. [62]
				WBF—no significant relationship	
				WBA—increased significantly (as proxy by body moving amplitude)	
Harris's hawk *Parabuteo unicinctus*	outdoor flight—accelerometery data and kinematic analysis using video recordings	climbing flight	—	*as climb power increased*	Van Walsum *et al*. [13]
				WBF—increased linearly with lesser variation	
				WBA—increased linearly with higher variation (as proxy by body moving amplitude)	
common swift *Apus apus*	wind tunnel—PIV and kinematics analysis with high-speed cameras	level flight	8–9.2	*as speed increased*	Henningsson *et al*. [63]
				WBF—decreased	
				WBA—increased	
ruby-throated hummingbird *Archilochus colubris*	flight experiments in an airtight cube—varying air density treated with heliox	hovering	—	*as power increased*	Chai & Dudley [64]
				WBF—increased (less significant)	
				WBA—increased (significantly)	
				*as air density decreased*	
				power—increased	
ruby-throated hummingbird *Archilochus colubris*	flight experiments in an airtight cube—varying air density treated with helium	hovering	—	*as power increased*	Chai & Dudley [65]
				WBF—did not vary	
				WBA—increased (significantly)	
				*as air density decreased*	
				power—increased	
ruby-throated hummingbird *Archilochus colubris*	cubic testing arena—surgical procedures to measure flight muscle activity and kinematics analysis with high-speed cameras	hovering	—	*as load lifted increased*	Mahalingam & Welch [66]
				WBF—did not vary	
				WBA—increased (significantly)	
				*as air density decreased*	
				WBF—did not vary	
				WBA—increased (significantly)	
rufous hummingbird *Selasphorus rufus*	wind tunnel—kinematics analysis with high-speed cameras	hovering and level flight	0–12	*as speed increased*	Tobalske *et al*. [67]
				WBF—did not vary	
				WBA—increased (approx. U shaped)	

Wingbeat frequency had a U-shaped relationship with speed (to a variable degree) in the following species: pigeon, barn swallow, thrush nightingale, zebra finch, budgerigar and Eurasian teal. However, two further studies with thrush nightingale and cockatiel found no/different relationships between wingbeat frequency and speed, and another three studies found no relationship between wingbeat frequency and speed in black-legged kittiwake, common swift and a rufous hummingbird (table 4). One study found a U-shaped relationship between wingbeat amplitude and speed, and three others found a notable positive relationship (in pigeons, kittiwake and common swift) (table 4). The two studies on pigeons found that wingbeat amplitude varied in ascending and descending flights. Information on wingbeat amplitude was available for the budgerigars and it did not vary significantly with speed. Four studies on hummingbirds showed that wingbeat amplitude increased with power during hovering.

Results from a further seven studies showed that the relationships between metabolic power and wingbeat frequency and amplitude were similarly variable (table 5). While wingbeat frequency was positively correlated with the metabolic power in four studies, either it did not vary significantly or stayed constant in two studies, and it declined in one study. Furthermore, cockatiels in two different studies exhibited a discrepancy between the wingbeat frequency and the power variation for the same speed range and flight mode: while the power had a U-shaped relationship with speed, wingbeat frequency varied in the same fashion in one case but was negatively related to speed in another. Out of three studies reported, wingbeat amplitude was closely correlated with power in two.

**Table 5.** Summary of studies assessing the relationship between wingbeat frequency, amplitude and metabolic power.

species	method	flight mode	speed (m s^−1^)	remarks	source
common starling *Sturnus vulgaris*	wind tunnel—measurements of oxygen consumption and carbon dioxide production, and kinematics analysis recorded on magnetic tape	burst flapping and gliding	6–18	*as power stayed almost constant*	Torre-Bueno & Larochelle [31]
				WBF—constant	
				WBA—varied approx. U shaped	
black-billed magpie *Pica hudsonia*	wind tunnel—pectoralis muscle force based on bone-strain recordings and muscle fibre length	hovering and level flight	0–14	*as power varied L shaped*	Dial *et al*. [68]
				WBF—varied U shaped	
cockatiel *Nymphicus hollandicus*	wind tunnel—measurements of oxygen consumption and carbon dioxide production	level flight	6–14	*as speed varied as U shaped*	Morris *et al*. [69]
				WBF—varied approx. U shaped	
				WBA—varied approx. U shaped	
cockatiels *Nymphicus hollandicus*	wind tunnel—measurement of oxygen consumption using masks	level flight	5–15	*as power varied as U shaped*	Bundle *et al*. [70]
				WBF—decreased significantly	
budgerigars *Melopsittacus undulatus*	wind tunnel—measurement of oxygen consumption using masks	level flight	5–15	*as power varied as U shaped*	Bundle *et al*. [70]
				WBF—did not vary significantly	
budgerigars *Melopsittacus undulatus*	wind tunnel—measurements of oxygen consumption and carbon dioxide production	ascending, level and descending flight	5–13	*as power varied as U shaped*	Tucker [71]
				WBF—constant	
bar-headed goose *Anser indicus*	migratory flight—measurements using data loggers	ascending, level and descending flight	—	power increased as WBF^6.96^	Bishop *et al*. [8]
				WBA increased with power	

## 4. Discussion

The total power output of a bird in flapping flight varies between level, accelerating, ascending/descending, manoeuvring and load carrying flight, as well as with flight speed. Birds are expected to modulate the power output predominantly through wingbeat frequency and/or wingbeat amplitude changes, as first principles state that power output is directly proportional to the cube of the product of wingbeat frequency and amplitude. Metrics from onboard accelerometers should be able to provide insight into the relative importance of both these parameters. Our data from wind tunnel flights confirm this, by showing that the amplitude of the dorsoventral body acceleration (heave) and the wingbeat amplitude are positively related within a wingbeat cycle. While the *R*^2^ values varied substantially between the two pigeons (0.08 versus 0.24; figure 3), this is unlikely to reflect differences in kinematics, which should be consistent across individuals for the same flight style; instead, the variance in these relationships is likely to have been affected by the flight consistency, and possibly the stability of the magnet attachment. More broadly, the ability to resolve relative changes in wingbeat amplitude from the acceleration signal may show some variation with flight style; for instance, peaks associated with wingbeats can be harder to resolve against a baseline that varies due to centripetal acceleration, as occurs throughout the dynamic soaring cycle. This may help explain the lack of a correlation between wingbeat frequency and acceleration amplitude in three of the four species that used dynamic soaring in this study, although this could also reflect a genuine absence of a relationship in this group.

The question that follows is, to what extent do birds modify wingbeat frequency and/or amplitude to modulate power output? We found that wingbeat frequency and amplitude were correlated for pigeons, and wingbeat amplitude increased with increasing flight speed (similar to another pigeon study [6]). It was, therefore, surprising that we found no relationship between wingbeat frequency, amplitude and airspeed in pigeons during homing flights. The discrepancy between our wind tunnel and ‘wild’ flights may be related to the extremely variable nature of pigeon homing flights when flying solo [3]. Indeed, the substantial (and costly) variation in speed and rate of change in altitude has been proposed to serve as a predator avoidance strategy, which birds such as pigeons may adopt when flocking is not possible [3]. This is relevant in the current context as it could mask a relationship between wingbeat frequency, amplitude and airspeed in homing flights. This highlights that birds experience very different biological and physical environments when flying in the laboratory and in the wild, which can in turn affect their kinematics. There are also likely to be errors in the estimation of airspeed, as wind conditions were recorded near the release site and while this was within 5.7 km of the loft, the wind field will be affected by the local topography as well as flight altitude. These errors will be larger for the tropicbird study, where GPS locations were recorded once a minute and wind speeds were measured up to tens of kilometres away from the bird locations, which likely contributes to the lack of a correlation between kinematic parameters and airspeed in this species. Nonetheless, the positive relationship between kinematic parameters and climb rates for tropicbirds shows that relationships can be resolved using high-frequency data from birds flying in the wild as, unlike wind, pressure was recorded with sub-second resolution.

Our finding that wingbeat frequency and amplitude were positively correlated in 11 of the 14 species that we investigated suggests that both parameters tend to be involved in power modulation across a range of morphologies and body masses. However, the low *R*^2^ values indicate that they are unlikely to covary in a straightforward manner, as also indicated by the variable relationship between wingbeat frequency and amplitude in other studies: five studies reported a positive correlation, five reported a negative relationship and three reported no correlation (table 2). Nonetheless, our review of the literature did suggest that birds tend to increase their wingbeat amplitude more in the most energetically demanding forms of flight (table 4), consistent with our finding that tropicbirds increased their wingbeat amplitude to a greater extent than frequency when climbing. For instance, while Usherwood *et al*. [6] found that wingbeat frequency increased during all flight modes for pigeons flying in a flock, the wingbeat amplitude increased with induced power, climb rate, and accelerating flight. Parallels can be found in studies by Tobalske & Biewener [30], where pigeons varied their wingbeat amplitude, but not frequency, during take-off and landing. Zebra finches (*Taeniopygia guttata*) were also found to modulate wingbeat amplitude rather than wingbeat frequency for high power events [20,60], but not in level flight [20]. Other studies have shown that wingbeat amplitude increased to meet the power demand associated with load carrying in hovering/vertical flight, whereas the wingbeat frequency remained near constant [66]. Similarly, hummingbirds increased their wingbeat amplitude when flying in low-density air, both in the laboratory [64–66] and in the field along natural elevational gradients [72,73], with wingbeat amplitudes up to 180° at flight failure densities.

Yet flight mode alone does not explain which kinematic parameter birds select to modulate their flight power, as while we found 10 studies where wingbeat frequency increased with airspeed in non-hovering flight, there were negative relationships between frequency and airspeed in two studies, and no relationship in 10 studies (table 4) including our ‘wild’ data. The variation across studies is striking and extends beyond comparisons between laboratory and field settings. In fact, differing relationships were found within two species (cockatiels and thrush nightingales) in experimental studies, which may indicate the influence of factors such as turbulence levels in wind tunnels, or the difficulties of training birds to maintain steady level flight, both of which could have a notable impact on the variability of kinematic parameters over fine scales.

We found limited support for the hypothesis that morphology influences variation in kinematic parameters, although birds with high residual wing loading, such as auks, did appear to have relatively low variation in wingbeat frequency, consistent with their relatively low available power. It would be interesting to see whether this non-significant negative correlation persists if data from a greater number of species were included.

This study has focused on variation in wingbeat frequency and amplitude. However, birds can also vary the aerodynamic forces through changes in the other wingbeat kinematic parameters and wing flexing and it is unclear whether and how they could all be captured by body-mounted accelerometers. Other kinematics parameters that have a significant role in power output include the upstroke-to-downstroke ratio, stroke-plane angle, span ratio, twist and angle of attack. In experiments with a house martin (*Delichon urbicum*) and a thrush nightingale (*Luscinia luscinia*), the upstroke-to-downstroke ratio and span ratio varied with increasing flight speed, whereas the wingbeat frequency and amplitude did not [25,26]. Similarly, Ward *et al*. [27] showed that for a common starling (*Sturnus vulgaris*), the wingbeat frequency and amplitude were the least important parameters associated with an increase in power, compared to variations in the stroke-plane angle and downstroke ratio. Finally, several species vary the body angle and stroke-plane angle to support weight at low speeds and augment thrust at higher speeds, while frequency and amplitude varied to a lesser degree in these scenarios [74]. The situation is potentially even more complex in intermittent flap-bounding flight, and indeed, cycle time spent flapping, flapping-and-bounding duration and the number of flaps were more important than wingbeat frequency and amplitude for a zebra finch increasing its flight speed [59].

Overall, in terms of the implications for acceleration metrics to act as proxies for flight power, it is clear that body-mounted accelerometers can provide information on wingbeat amplitude as well as frequency, both of which show substantial variation when considered across free-ranging flights in multiple species. Acceleration metrics that incorporate variation due to wingbeat frequency and amplitude, such as DBA and body power [34,36] should, therefore, be more robust proxies for power use than wingbeat frequency alone. In support of this, DBA has been shown to be a better predictor of overall energy expenditure (estimated with doubly labelled water) than flight time or wingbeat frequency in auks [35,75]. Nonetheless, wingbeat frequency and amplitude are only partial determinants of the wingbeat kinematics associated with power, and other factors play a substantial role in power production for certain flight types [76]. Some of these, e.g. the downstroke ratio, may be estimated from onboard accelerometers [14], although the magnetometer is a valuable addition in this regard, highlighting when the downstroke begins and ends (e.g. figure 2). Beyond this, what is clear is that while relationships between DBA and energy expenditure are linear for terrestrial and aquatic forms of locomotion (a relationship that holds across tens of species and over different timeframes [33,77,78]), it is unlikely to be the case for all types of flight, not least because of the varying contribution of wingbeat frequency and amplitude to power. Experiments with independent estimates of power output will provide further insight into the performance of acceleration-based proxies and the extent to which single metrics applied across species and contexts.

## Ethics

We have included the details of ethical approval for each species in the electronic supplementary material [79].

## Data Availability

Additional data are available in the electronic supplementary material [79].

## References

[1] Kranstauber B, Weinzierl R, Wikelski M, Safi K. 2015 Global aerial flyways allow efficient travelling. Ecol. Lett. **18**, 1338-1345. (10.1111/ele.12528) 26477348

[2] Weimerskirch H, Louzao M, De Grissac S, Delord K. 2012 Changes in wind pattern alter albatross distribution and life-history traits. Science **335**, 211-214. (10.1126/science.1210270) 22246774

[3] Garde B et al. 2021 Fine-scale changes in speed and altitude suggest protean movements in homing pigeon flights. R. Soc. Open Sci. **8**, 210130. (10.1098/rsos.210130) 34017602 PMC8131938

[4] Portugal SJ et al. 2014 Upwash exploitation and downwash avoidance by flap phasing in ibis formation flight. Nature **505**, 399-402. (10.1038/nature12939) 24429637

[5] Sankey DWE, Portugal SJ. 2019 When flocking is costly: reduced cluster-flock density over long-duration flight in pigeons. Sci. Nat. **106**, 47. (10.1007/s00114-019-1641-x) 31309338

[6] Usherwood JR, Stavrou M, Lowe JC, Roskilly K, Wilson AM. 2011 Flying in a flock comes at a cost in pigeons. Nature **474**, 494-497. (10.1038/nature10164) 21697946 PMC3162477

[7] Hicks O, Burthe S, Daunt F, Butler A, Bishop C, Green JA. 2017 Validating accelerometry estimates of energy expenditure across behaviours using heart rate data in a free-living seabird. J. Exp. Biol. **220**, 1875-1881. (10.1242/jeb.152710) 28258086 PMC5450806

[8] Bishop CM et al. 2015 The roller coaster flight strategy of bar-headed geese conserves energy during Himalayan migrations. Science **347**, 250-254. (10.1126/science.1258732) 25593180

[9] Furness RW, Bryant DM. 1996 Effect of wind on field metabolic rates of breeding northern fulmars. Ecology **77**, 1181-1188. (10.2307/2265587)

[10] Sapir N, Wikelski M, McCue MD, Pinshow B, Nathan R. 2010 Flight modes in migrating European bee-eaters: heart rate may indicate low metabolic rate during soaring and gliding. PLoS ONE **5**, e13956. (10.1371/journal.pone.0013956) 21085655 PMC2978710

[11] Cochran WW, Bowlin MS, Wikelski M. 2008 Wingbeat frequency and flap-pause ratio during natural migratory flight in thrushes. Integr. Comp. Biol. **48**, 134-151. (10.1093/icb/icn044) 21669779

[12] Sato K, Daunt F, Watanuki Y, Takahashi A, Wanless S. 2008 A new method to quantify prey acquisition in diving seabirds using wing stroke frequency. J. Exp. Biol. **211**, 58-65. (10.1242/jeb.009811) 18083733

[13] Van Walsum TA, Perna A, Bishop CM, Murn CP, Collins PM, Wilson RP, Halsey LG. 2020 Exploring the relationship between flapping behaviour and accelerometer signal during ascending flight, and a new approach to calibration. Ibis **162**, 13-26. (10.1111/ibi.12710)

[14] Taylor LA, Taylor GK, Lambert B, Walker JA, Biro D, Portugal SJ. 2019 Birds invest wingbeats to keep a steady head and reap the ultimate benefits of flying together. PLoS Biol. **17**, e3000299. (10.1371/journal.pbio.3000299) 31211769 PMC6581236

[15] Engel S, Bowlin MS, Hedenström A. 2010 The role of wind-tunnel studies in integrative research on migration biology. Integr. Comp. Biol. **50**, 323-335. (10.1093/icb/icq063) 21558207

[16] Hedenström A, Lindström Å. 2017 Wind tunnel as a tool in bird migration research. J. Avian Biol. **48**, 37-48. (10.1111/jav.01363)

[17] Norberg UM. 2012 Vertebrate flight: mechanics, physiology, morphology, ecology and evolution, vol. 27. Berlin, Germany: Springer Science & Business Media.

[18] Pennycuick CJ. 2008 Modelling the flying bird. London, UK: Elsevier.

[19] Tobalske BW, Hedrick TL, Dial KP, Biewener AA. 2003 Comparative power curves in bird flight. Nature **421**, 363-366. (10.1038/nature01284) 12540899

[20] Ellerby DJ, Askew GN. 2007 Modulation of pectoralis muscle function in budgerigars *Melopsitaccus undulatus* and zebra finches *Taeniopygia guttata* in response to changing flight speed. J. Exp. Biol. **210**, 3789-3797. (10.1242/jeb.006296) 17951420

[21] Hedrick TL, Tobalske BW, Biewener AA. 2003 How cockatiels (*Nymphicus hollandicus*) modulate pectoralis power output across flight speeds. J. Exp. Biol. **206**, 1363-1378. (10.1242/jeb.00272) 12624171

[22] Pennycuick CJ, Klaassen M, Kvist A, Lindström Å. 1996 Wingbeat frequency and the body drag anomaly: wind-tunnel observations on a thrush nightingale (*Luscinia luscinia*) and a teal (*Anas crecca*). J. Exp. Biol. **199**, 2757-2765. (10.1242/jeb.199.12.2757) 9320660

[23] Schmidt-Wellenburg CA, Biebach H, Daan S, Visser GH. 2007 Energy expenditure and wing beat frequency in relation to body mass in free flying barn swallows (*Hirundo rustica*). J. Comp. Physiol. B **177**, 327-337. (10.1007/s00360-006-0132-5) 17171355

[24] Pennycuick CJ, Hedenström A, Rosén M. 2000 Horizontal flight of a swallow (*Hirundo rustica*) observed in a wind tunnel, with a new method for directly measuring mechanical power. J. Exp. Biol. **203**, 1755-1765. (10.1242/jeb.203.11.1755) 10804165

[25] Rosén M, Spedding GR, Hedenstrom A. 2004 The relationship between wingbeat kinematics and vortex wake of a thrush nightingale. J. Exp. Biol. **207**, 4255-4268. (10.1242/jeb.01283) 15531647

[26] Rosén M, Spedding GR, Hedenström A. 2007 Wake structure and wingbeat kinematics of a house-martin *Delichon urbica*. J. R. Soc. Interface **4**, 659-668. (10.1098/rsif.2007.0215) 17264054 PMC2373391

[27] Ward S, Möller U, Rayner JMV, Jackson DM, Bilo D, Nachtigall W, Speakman JR. 2001 Metabolic power, mechanical power and efficiency during wind tunnel flight by the European starling *Sturnus vulgaris*. J. Exp. Biol. **204**, 3311-3322. (10.1242/jeb.204.19.3311) 11606605

[28] Shyy W, Aono H, Chimakurthi SK, Trizila P, Kang CK, Cesnik CES, Liu H. 2010 Recent progress in flapping wing aerodynamics and aeroelasticity. Prog. Aerosp. Sci. **46**, 284-327. (10.1016/j.paerosci.2010.01.001)

[29] Floryan D, Van Buren T, Smits AJ. 2018 Efficient cruising for swimming and flying animals is dictated by fluid drag. Proc. Natl Acad. Sci. USA **115**, 8116-8118. (10.1073/pnas.1805941115) 29915088 PMC6094110

[30] Tobalske BW, Biewener AA. 2008 Contractile properties of the pigeon supracoracoideus during different modes of flight. J. Exp. Biol. **211**, 170-179. (10.1242/jeb.007476) 18165244

[31] Torre-Bueno JR, Larochelle J. 1978 The metabolic cost of flight in unrestrained birds. J. Exp. Biol. **75**, 223-229. (10.1242/jeb.75.1.223) 702041

[32] Wang Y, Yin Y, Ge S, Li M, Zhang Q, Li J, Wu Y, Li D, Dudley R. 2019 Limits to load-lifting performance in a passerine bird: the effects of intraspecific variation in morphological and kinematic parameters. PeerJ **7**, e8048. (10.7717/peerj.8048) 31741797 PMC6858814

[33] Wilson RP et al. 2020 Estimates for energy expenditure in free-living animals using acceleration proxies: a reappraisal. J. Anim. Ecol. **89**, 161-172. (10.1111/1365-2656.13040) 31173339 PMC7030956

[34] Wilson RP, White CR, Quintana F, Halsey LG, Liebsch N, Martin GR, Butler PJ. 2006 Moving towards acceleration for estimates of activity-specific metabolic rate in free-living animals: the case of the cormorant. J. Anim. Ecol. **75**, 1081-1090. (10.1111/j.1365-2656.2006.01127.x) 16922843

[35] Elliott KH, Le Vaillant M, Kato A, Speakman JR, Ropert-Coudert Y. 2013 Accelerometry predicts daily energy expenditure in a bird with high activity levels. Biol. Lett. **9**, 20120919. (10.1098/rsbl.2012.0919) 23256182 PMC3565507

[36] Spivey RJ, Bishop CM. 2013 Interpretation of body-mounted accelerometry in flying animals and estimation of biomechanical power. J. R. Soc. Interface **10**, 20130404. (10.1098/rsif.2013.0404) 23883951 PMC3758002

[37] Wilson R, Liebsch N. 2003 Up-beat motion in swinging limbs: new insights into assessing movement in free-living aquatic vertebrates. Mar. Biol. **142**, 537-547. (10.1007/S00227-002-0964-9)

[38] Pennycuick CJ, Alerstam T, Hedenström A. 1997 A new low-turbulence wind tunnel for bird flight experiments at Lund University, Sweden. J. Exp. Biol. **200**, 1441-1449. (10.1242/jeb.200.10.1441) 9319339

[39] Orben RA, Paredes R, Roby DD, Irons DB, Shaffer SA. 2015 Body size affects individual winter foraging strategies of thick-billed murres in the Bering Sea. J. Anim. Ecol. **84**, 1589-1599. (10.1111/1365-2656.12410) 26095664

[40] Spear LB, Ainley DG. 1997 Flight behaviour of seabirds in relation to wind direction and wing morphology. Ibis **139**, 221-233. (10.1111/j.1474-919x.1997.tb04620.x)

[41] Warham J. 1977 Wing loadings, wing shapes, and flight capabilities of Procellariiformes. N. Z. J. Zool. **4**, 73-83. (10.1080/03014223.1977.9517938)

[42] Pennycuick C. 1997 Actual and ‘optimum’ flight speeds: field data reassessed. J. Exp. Biol. **200**, 2355-2361. (10.1242/jeb.200.17.2355) 9320274

[43] Quintana F, Wilson R, Dell'Arciprete P, Shepard E, Laich AG. 2011 Women from Venus, men from Mars: inter-sex foraging differences in the imperial cormorant *Phalacrocorax atriceps* a colonial seabird. Oikos **120**, 350-358. (10.1111/j.1600-0706.2010.18387.x)

[44] Phillips RA, Silk JRD, Phalan B, Catry P, Croxall JP. 2004 Seasonal sexual segregation in two *Thalassarche* albatross species: competitive exclusion, reproductive role specialization or foraging niche divergence? Proc. Natl Acad. Sci. USA **271**, 1283-1291. (10.1098/rspb.2004.2718) PMC1691717 15306353

[45] Shirai M, Niizuma Y, Tsuchiya K, Yamamoto M, Oka N. 2013 Sexual size dimorphism in streaked shearwaters *Calonectris leucomelas*. Ornithol. Sci. **12**, 57-62. (10.2326/osj.12.57)

[46] Hentze NT. 2012 Characteristics of over-ocean flocking by Pacific dunlins (*Calidris alpina pacifica*). MSc thesis, Biological Sciences Department, SFU, Canada.

[47] Garde B et al. 2022 Ecological inference using data from accelerometers needs careful protocols. Methods Ecol. Evol. **13**, 813-825. (10.1111/2041-210X.13804) 35910299 PMC9303593

[48] Lee SY, Scott GR, Milsom WK. 2008 Have wing morphology or flight kinematics evolved for extreme high altitude migration in the bar-headed goose? Comp. Biochem. Physiol. C: Toxicol. Pharmacol. **148**, 324-331. (10.1016/j.cbpc.2008.05.009) 18635402

[49] Wilson RP, Pütz K, Peters G, Culik B, Scolaro JA, Charrassin J-B, Ropert-Coudert Y. 1997 Long-term attachment of transmitting and recording devices to penguins and other seabirds. Wildl. Soc. Bull. **25**, 101-106.

[50] Biro D, Guilford T, Dell'Omo G, Lipp HP. 2002 How the viewing of familiar landscapes prior to release allows pigeons to home faster: evidence from GPS tracking. J. Exp. Biol. **205**, 3833-3844. (10.1242/jeb.205.24.3833) 12432007

[51] Shepard E et al. 2008 Identification of animal movement patterns using tri-axial accelerometry. Endanger. Species Res. **10**, 47-60. (10.3354/esr00084)

[52] R Core Team. 2020 R: a language and environment for statistical computing. Vienna, Austria: R Foundation for Statistical Computing. See http://www.r-project.org/index.html.

[53] Pinheiro J, Bates D, DebRoy S, Sarkar D, Heisterkamp S, Van Willigen B, Maintainer R. 2017 Package ‘nlme’. Linear and nonlinear mixed effects models, version 3(1). See https://svn.r-project.org/R-packages/trunk/nlme/.

[54] Barton K, Barton MK. 2015 Package ‘MuMIn’. Version 1, 18. See https://cran.r-project.org/package=MuMIn.

[55] Delignette-Muller ML, Dutang C, Pouillot R, Denis J-B. 2015 Package fitdistrplus. Vienna, Austria: R Foundation for Statistical Computing.

[56] Crandell KE, Tobalske BW. 2011 Aerodynamics of tip-reversal upstroke in a revolving pigeon wing. J. Exp. Biol. **214**, 1867-1873. 21562173 10.1242/jeb.051342

[57] Bilo D, Lauck A, Nachtigall W. 1984 Measurement of linear body accelerations and calculation of the instantaneous aerodynamic lift and thrust in a pigeon flying in a wind tunnel. Biona-Report **3**, 87-108.

[58] McFarlane L, Altringham JD, Askew GN. 2016 Intra-specific variation in wing morphology and its impact on take-off performance in blue tits (*Cyanistes caeruleus*) during escape flights. J. Exp. Biol. **219**, 1369-1377. (10.1242/jeb.126888) 26994175 PMC4874562

[59] Tobalske BW, Peacock WL, Dial KP. 1999 Kinematics of flap-bounding flight in the zebra finch over a wide range of speeds. J. Exp. Biol. **202**, 1725-1739. (10.1242/jeb.202.13.1725) 10359676

[60] Bahlman JW, Baliga VB, Altshuler DL. 2020 Flight muscle power increases with strain amplitude and decreases with cycle frequency in zebra finches (*Taeniopygia guttata*). J. Exp. Biol. **223**, jeb225839. (10.1242/jeb.225839) 33046567

[61] Morris CR, Askew GN. 2010 The mechanical power output of the pectoralis muscle of cockatiel (*Nymphicus hollandicus*): the in vivo muscle length trajectory and activity patterns and their implications for power modulation. J. Exp. Biol. **213**, 2770-2780. (10.1242/jeb.035691) 20675547

[62] Collins PM, Green JA, Elliott KH, Shaw PJA, Chivers L, Hatch SA, Halsey LG. 2020 Coping with the commute: behavioural responses to wind conditions in a foraging seabird. J. Avian Biol. **51**, e02057. (10.1111/jav.02057)

[63] Henningsson P, Spedding GR, Hedenstrom A. 2008 Vortex wake and flight kinematics of a swift in cruising flight in a wind tunnel. J. Exp. Biol. **211**, 717-730. (10.1242/jeb.012146) 18281334

[64] Chai P, Dudley R. 1995 Limits to vertebrate locomotor energetics suggested by hummingbirds hovering in heliox. Nature **377**, 722-725. (10.1038/377722a0)

[65] Chai P, Dudley R. 1996 Limits to flight energetics of hummingbirds hovering in hypodense and hypoxic gas mixtures. J. Exp. Biol. **199**, 2285-2295. (10.1242/jeb.199.10.2285) 8896366

[66] Mahalingam S, Welch Jr KC. 2013 Neuromuscular control of hovering wingbeat kinematics in response to distinct flight challenges in the ruby-throated hummingbird, *Archilochus colubris*. J. Exp. Biol. **216**, 4161-4171. (10.1242/jeb.089383) 23948477

[67] Tobalske BW, Warrick DR, Clark CJ, Powers DR, Hedrick TL, Hyder GA, Biewener AA. 2007 Three-dimensional kinematics of hummingbird flight. J. Exp. Biol. **210**, 2368-2382. (10.1242/jeb.005686) 17575042

[68] Dial KP, Biewener AA, Tobalske BW, Warrick DR. 1997 Mechanical power output of bird flight. Nature **390**, 67-70. (10.1038/36330)

[69] Morris CR, Nelson FE, Askew GN. 2010 The metabolic power requirements of flight and estimations of flight muscle efficiency in the cockatiel (*Nymphicus hollandicus*). J. Exp. Biol. **213**, 2788-2796. (10.1242/jeb.035717) 20675549

[70] Bundle MW, Hansen KS, Dial KP. 2007 Does the metabolic rate-flight speed relationship vary among geometrically similar birds of different mass? J. Exp. Biol. **210**, 1075-1083. (10.1242/jeb.02727) 17337719

[71] Tucker BYVA. 1968 Respiratory exchange and evaporative water loss in the flying budgerigar. J. Exp. Biol. **48**, 67-87. (10.1242/jeb.48.1.67)

[72] Altshuler DL, Dudley R. 2006 The physiology and biomechanics of avian flight at high altitude. Integr. Comp. Biol. **46**, 62-71. (10.1093/icb/icj008) 21672723

[73] Altshuler DL, Dudley R. 2003 Kinematics of hovering hummingbird flight along simulated and natural elevational gradients. J. Exp. Biol. **206**, 3139-3147. (10.1242/jeb.00540) 12909695

[74] Tobalske BW, Dial KP. 1996 Flight kinematics of black-billed magpies and pigeons over a wide range of speeds. J. Exp. Biol. **199**, 263-280. (10.1242/jeb.199.2.263) 9317775

[75] Elliott KH et al. 2014 Windscapes shape seabird instantaneous energy costs but adult behavior buffers impact on offspring. Mov. Ecol. **2**, 17. (10.1186/s40462-014-0017-2) 26019870 PMC4445632

[76] Berg AM, Biewener AA. 2008 Kinematics and power requirements of ascending and descending flight in the pigeon (*Columba livia*). J. Exp. Biol. **211**, 1120-1130. (10.1242/jeb.010413) 18344487

[77] Halsey LG, Shepard ELC, Quintana F, Gomez Laich A, Green JA, Wilson RP. 2009 The relationship between oxygen consumption and body acceleration in a range of species. Comp. Biochem. Physiol. A Mol. Integr. Physiol. **152**, 197-202. (10.1016/j.cbpa.2008.09.021) 18854225

[78] Halsey LG, Shepard ELC, Wilson RP. 2011 Assessing the development and application of the accelerometry technique for estimating energy expenditure. Comp. Biochem. Physiol. A Mol. Integr. Physiol. **158**, 305-314. (10.1016/j.cbpa.2010.09.002) 20837157

[79] Krishnan K et al. 2022 The role of wingbeat frequency and amplitude in flight power. Figshare. (10.6084/m9.figshare.c.6145476) PMC9403799 36000229

